# Comparing oncologic and surgical outcomes of robotic and laparoscopic pancreatoduodenectomy in patients with pancreatic cancer: a propensity-matched analysis

**DOI:** 10.1007/s00464-024-10783-1

**Published:** 2024-03-18

**Authors:** Chase J. Wehrle, Jenny H. Chang, Abby R. Gross, Kimberly Woo, Robert Naples, Kathryn A. Stackhouse, Fadi Dahdaleh, Toms Augustin, Daniel Joyce, Robert Simon, R. Matthew Walsh, Samer A. Naffouje

**Affiliations:** 1 Department of General Surgery, Cleveland Clinic Foundation, 9500 Euclid Avenue, Cleveland, OH 44195 USA; 2 Department of Surgical Oncology, Edward-Elmhurst Health, Naperville, IL USA

**Keywords:** Pancreatoduodenectomy, Minimally invasive surgery, Pancreatic ductal adenocarcinoma, Robotic surgery, Laparoscopic surgery

## Abstract

**Introduction:**

Minimally invasive Pancreatoduodenectomy (MIPD), or the Whipple procedure, is increasingly utilized. No study has compared laparoscopic (LPD) and robotic (RPD) approaches, and the impact of the learning curve on oncologic, technical, and post-operative outcomes remains relatively understudied.

**Methods:**

The National Cancer Database was queried for patients undergoing LPD or RPD from 2010 to 2020 with a diagnosis of pancreatic cancer. Outcomes were compared between approaches using propensity-score matching (PSM); the impact of annual center-level volume of MIPD was also assessed by dividing volume into quartiles.

**Results:**

A total of 3,342 patients were included. Most (*n* = 2,716, 81.3%) underwent LPD versus RPD (*n* = 626, 18.7%). There was a high rate (20.2%, *n* = 719) of positive margins. Mean length-of-stay (LOS) was 10.4 ± 8.9 days. Thirty-day mortality was 2.8% (*n* = 92) and ninety-day mortality was 5.7% (*n* = 189).

PSM matched 625 pairs of patients receiving LPD or RPD. After PSM, there was no differences between groups based on age, sex, race, CCI, T-stage, neoadjuvant chemo/radiotherapy, or type of PD. After PSM, there was a higher rate of conversion to open (HR = 0.68, 95%CI = 0.50–0.92)., but there was no difference in LOS (HR = 1.00, 95%CI = 0.92–1.11), 30-day readmission (HR = 1.08, 95% CI = 0.68–1.71), 30-day (HR = 0.78, 95% CI = 0.39–1.56) or 90-day mortality (HR = 0.70, 95% CI = 0.42–1.16), ability to receive adjuvant therapy (HR = 1.15, 95% CI = 0.92–1.44), nodal harvest (HR = 1.01, 95%CI = 0.94–1.09) or positive margins (HR = 1.19, 95% CI = 0.89–1.59).

Centers in lower quartiles of annual volume of MIPD demonstrated reduced nodal harvest (*p* = 0.005) and a higher rate of conversion to open (*p* = 0.038). Higher-volume centers had a shorter LOS (*p* = 0.012), higher rate of initiation of adjuvant therapy (*p* = 0.042), and, most strikingly, a reduction in 90-day mortality (*p* = 0.033).

**Conclusion:**

LPD and RPD have similar surgical and oncologic outcomes, with a lower rate of conversion to open in the robotic cohort. The robotic technique does not appear to eliminate the “learning curve”, with higher volume centers demonstrating improved outcomes, especially seen at minimum annual volume of 5 cases.

The pancreaticoduodenectomy (PD), or Whipple, has long been the standard of care for the surgical management of pancreatic head ductal adenocarcinoma since it was first described for this purpose in 1934 [1–4]. Since its’ inception, this has been primarily an open procedure, and was actually first described as a sequence of two open operations by Whipple [3, 4]. The first minimally invasive Pancreatoduodenectomy was performed laparoscopically in 1994 by Michael Gagner as treatment for pancreatitis [5]. He subsequently published a case series declaring that the laparoscopic PD (LPD) was feasible but noted that “no benefit seemed to be derived” [6].The first robotic PD (RPD) was performed in 2003 by Giulianotti [7, 8].

Both the feasibility and relative advantage of the minimally invasive PD (MIPD) are still debated. The rise of robotic surgery with the relative ergonomic benefits have only strengthened this debate [8, 9]. Multiple retrospective studies to date have demonstrated the feasibility and safety of the LPD and RPD procedures, and there have been significant comparisons of the LPD or MIPD to the open (OPD) technique [10, 11]. Generally speaking, the MIPD is associated with reduced length-of-stay (LOS) compared with the open approach at the expense of longer operative times, though continuously improving minimally invasive surgical techniques may improve this in the future [11]. However, data comparing approaches to MIPD (LPD vs RPD) are lacking.

We aim to compare laparoscopic and robotic Pancreatoduodenectomy using a large, nationwide database, both with respect to perioperative surgical outcomes and short-term-surrogates for oncologic success. To our knowledge, this represents the largest study to conduct this analysis to date, and the first to do so using a propensity-matched approach.

## Methods

This retrospective cohort study utilized data from the National Cancer Database (NCDB) to compare the outcomes of laparoscopic (LPD) and robotic Pancreatoduodenectomy (RPD). The NCDB is a national database sources from hospital registry sourced from 1,500 Commission on Cancer hospitals. The NCDB 2022 database was queried for patients undergoing LPD or RPD from 2010 to 2020. This range in time was chosen for their minimum two-year follow-up, which we deemed necessary to report adequate follow-up on chosen outcomes. Additional inclusion criteria were patients aged ≥ 18 years, and patients with a diagnosis of malignancy, commensurate with our data source. Exclusion criteria included patients who underwent open or planned hybrid Pancreatoduodenectomy, who underwent PD in conjunction with another procedure, patients aged < 18 years, or those who did not have a diagnosis of pancreatic adenocarcinoma. This study was approved by our institutional review board (IRB) prior to initiation.

Available NCDB data were queried for demographics and comorbidities including the Charlson comorbidity index. Oncologic information was obtained including histologic diagnosis, presenting stage, neoadjuvant therapies, pathologic outcomes, lymph node harvest, and adjuvant therapies. Surgical information included initial approach, conversion rate, and type of RPD (classic, pylorus-preserving [PPPD], extended). Finally, center-level data were recorded, including facility-type, facility location (metropolitan, urban, rural), and annual center volume.

The primary outcome of interest was complications and surgical outcomes between approaches (LPD vs RPD). Additional secondary outcomes included surgical-oncologic outcomes such as nodal harvest and positive margins. We finally sought to assess the impact of experience, as our assessment of the surgeon learning curve, on outcomes.

### Statistical analysis

Descriptive statistics were used to summarize patient demographics, oncologic information, and surgical variables. Categorical variables were presented as frequencies and percentages and continuous variables were presented as means with standard deviations or medians with interquartile range as appropriate. Groups were compared first using chi-squared tests for categorical variables or Mann–Whitney *U* tests for continuous variables. Conditional univariable logistic-regression was used to compare categorical outcomes and linear mixed effect modeling to compare continuous outcomes between LPD and RPD. These results are shown in Fig. 2. For comparison of outcomes between MIPD quartiles, we used Chi-square test of independence for categorial variables, and Kruskal–Wallis H test. Multivariate logistic-regression models were used assessing the impact of surgical approach on outcomes and measures of surgical success.

Propensity-score matching (PSM) was performed to account for potential confounding variables between the LPD and RPD cohorts. Matching was performed between groups in a 1:1 fashion. PSM utilized the nearest neighbor method per propensity scores with a caliper width of 0.05. Groups were matched on Age, Sex, Race, CCI, T-Stage, Neoadjuvant therapy, and type of PD (Classic, PPPD, or Extended). Subsequently, the comparison between LPD and RPD groups, pre- and post-matching, was done using multivariable conditional logistic regression. The model was built based on the patients’ likelihood of receiving LPD vs RPD based on their clinical and demographic profiles which include all listed variables in Table 2. No other variables were omitted or eliminated from the model. This is to ensure, to our best ability, that differences in postoperative outcomes are indeed attributed to the surgical approach, and not to a preexisting factor that could’ve biased the surgeon’s choice of the operative approach. SPSS v29 (IBM Corp., Armonk, NY) was used for the statistical analysis.

## Results

**Fig. 1:**
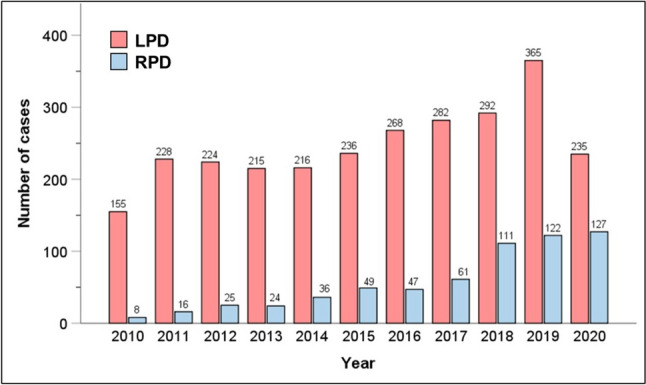
Chronological trends of LPD and RPD during the study period of 2010–2020. *LPD* laparoscopic pancreatoduodenectomy, *RPD* robotic pancreatoduodenectomy

A total of 3,342 patients met inclusion criteria from 2010 to 2020. The majority (*n* = 2716, 81.3%) underwent LPD versus RPD (*n* = 626, 18.7%). Most patients were male (*n* = 1720, 51.5%), white (*n* = 2678, 80.1%), and CCI 0 (*n* = 2165, 64.8%). The majority of MIPD’s were performed in Academic/Research Program settings (A/RP, *n* = 1884, 56.4%) versus Comprehensive Community Cancer Programs (CCCP, *n* = 725, 21.7%), Integrated Network Cancer Programs (INCP, *n* = 664, 19.9%), or Community Cancer Programs (CCC, *n* = 41, 1.2%). The median overall volume of PD by any approach was 17 (IQR 8–32) and of MIPD was 4 (IQR 2–10). Most (*n* = 2669, 79.9%) were performed in urban settings [Table 1]. The number of cases of RPD has increased nearly every year from 2010 to 2020. There was a general increase in rates of LPD year-to-year until 2020, when there was a drop in case volume (Fig. 1).

**Table 1:** Demographic and clinical characteristics of selected patients with pancreatic adenocarcinoma in the head of pancreas who underwent laparoscopic or robotic PD with curative intent between 2010 and 2020

N		3342
Age	Mean ± SD, median	66.4 ± 10.2, 67
Sex	Male	1720 (51.5%)
	Female	1622 (48.5%)
Race	White	2678 (80.1%)
	Black	321 (9.6%)
	Other	343 (10.3%)
Charlson score	0	2165 (64.8%)
	1	858 (25.7%)
	2	211 (6.3%)
	3	108 (3.2%)
T-stage	T1	581 (17.4%)
	T2	922 (27.6%)
	T3	1839 (55.0%)
Neoadjuvant therapy	Chemotherapy	1148 (34.4%)
	Radiation	362 (10.8%)
Facility type	CCC	41 (1.2%)
	CCCP	725 (21.7%)
	A/RP	1884 (56.4%)
	INCP	664 (19.9%)
	Not reported	28 (0.8%)
Annual PD center volume	Median [IQR]	17 [8–32]
Annual MIPD center volume	Median [IQR]	4 [2–10]
Area	Metropolitan	2,669 (79.9%)
	Urban	423 (12.7%)
	Rural	48 (1.4%)
	Not reported	202 (6.0%)
Procedure	Classic PD	2,632 (78.8%)
	Pylorus-preserving PD	417 (12.5%)
	Extended PD	293 (8.8%)
Approach	Laparoscopic PD	2,716 (81.3%)
	Robotic PD	626 (18.7%)
Conversion	No	2,623 (78.5%)
	Yes	719 (21.5%)
Margins	Negative	2,666 (79.8%)
	Positive	676 (20.2%)
Examined nodes	Mean ± SD, median	19.3 ± 9.9, 18
Length-of-stay	Mean ± SD, median	10.4 ± 8.9, 8
Unplanned 30-day readmission		223 (6.7%)
Mortality	30-day	92 (2.8%)
	90-day	189 (5.7%)
Adjuvant systemic therapy		1,834 (54.9%)

*A/RP* academic/research program, *CCC* community cancer program, *CCCP* comprehensive community cancer program, *INCP* integrated network cancer program, *IQR* interquartile range, *PD* pancreaticoduodenectomy, *SD* standard deviation

The overall rate of conversion to open across all included cases was 21.5% (*n* = 719). In all cases, there was a high rate (20.2%, *n* = 719) of positive margins on final pathology. Mean length-of-stay (LOS) was 10.4 ± 8.9 days. There was a relatively low (6.7%, *n* = 223) rate of unplanned readmission. Overall, 30- and 90-day mortality were 2.8% (*N* = 92) and 5.7% *n* = 189), respectively.

Before PSM, there was a higher rate of early-stage tumors in the RPD group (T1: 22.4% vs 16.2%, T2: 36.9% vs 25.4%, T3: 40.7% vs 58.3%, *p* < 0.001), higher rate of neoadjuvant chemo (40.9%, *n* = 256 vs 32.8%, *n* = 892, *p* < 0.001) and lower rate of radiotherapy (8.5%, *n* = 53 vs 11.4%, *n* = 308, *p* = 0.035). There was also a higher rate of non-classic PD (PPPD or Extended) in the LPD group before matching (*p* = 0.003) (Table 2). Propensity matching was performed matching 625 RPD patients to 625 patients receiving LPD. After PSM, there were no differences between groups based on age, sex, race, CCI, T-stage, neoadjuvant chemo/radiotherapy, or type of PD (Table 2).

**Table 2:** Comparison of baseline characteristics between the unmatched and 1:1 matched LPD and RPD patients

	Unmatched dataset			Matched dataset 1:1		
	LPD	RPD	*p*	LPD	RPD	*p*
N	2716	626		625	625	
Age	66.4 ± 10.1	66.5 ± 10.4	0.107	65.6 ± 10.1	66.5 ± 10.4	0.151
Sex			0.872			0.610
Male	1396 (51.4%)	324 (51.8%)		332 (53.1%)	323 (51.7%)	
Female	1,320 (48.6%)	302 (48.2%)		293 (46.9%)	302 (48.3%)	
Race			0.108			0.931
White	2166 (79.7%)	512 (81.8%)		506 (81.0%)	511 (81.8%)	
Black	257 (9.5%)	64 (10.2%)		66 (10.6%)	64 (10.2%)	
Other	293 (10.8%)	50 (8.0%)		53 (8.5%)	50 (8.0%)	
Charlson score			0.890			0.749
0	1763 (64.9%)	402 (64.2%)		398 (63.7%)	402 (64.3%)	
1	699 (25.7%)	159 (25.4%)		160 (25.6%)	159 (25.4%)	
2	168 (6.2%)	43 (6.9%)		39 (6.2%)	43 (6.9%)	
3	86 (3.2%)	22 (3.5%)		28 (4.5%)	21 (3.4%)	
T-stage			< 0.001*			0.760
T1	441 (16.2%)	140 (22.4%)		139 (22.2%)	140 (22.4%)	
T2	691 (25.4%)	231 (36.9%)		219 (35.0%)	230 (36.8%)	
T3	1584 (58.3%)	255 (40.7%)		267 (42.7%)	255 (40.8%)	
Neoadjuvant therapy						
Chemotherapy	892 (32.8%)	256 (40.9%)	< 0.001*	265 (42.4%)	255 (40.8%)	0.566
Radiation	309 (11.4%)	53 (8.5%)	0.035*	52 (8.3%)	53 (8.5%)	0.919
Pancreaticoduodenectomy			0.003*			0.496
Classic	2108 (77.6%)	524 (83.7%)		536 (85.8%)	523 (83.7%)	
Pylorus-preserving	361 (13.3%)	56 (8.9%)		45 (7.2%)	56 (9.0%)	
Extended	247 (9.1%)	46 (7.3%)		44 (7.0%)	46 (7.4%)	

*LPD* laparoscopic pancreaticoduodenectomy, *RPD* robotic pancreaticoduodenectomy

*Statistically significant

**Fig. 2:**
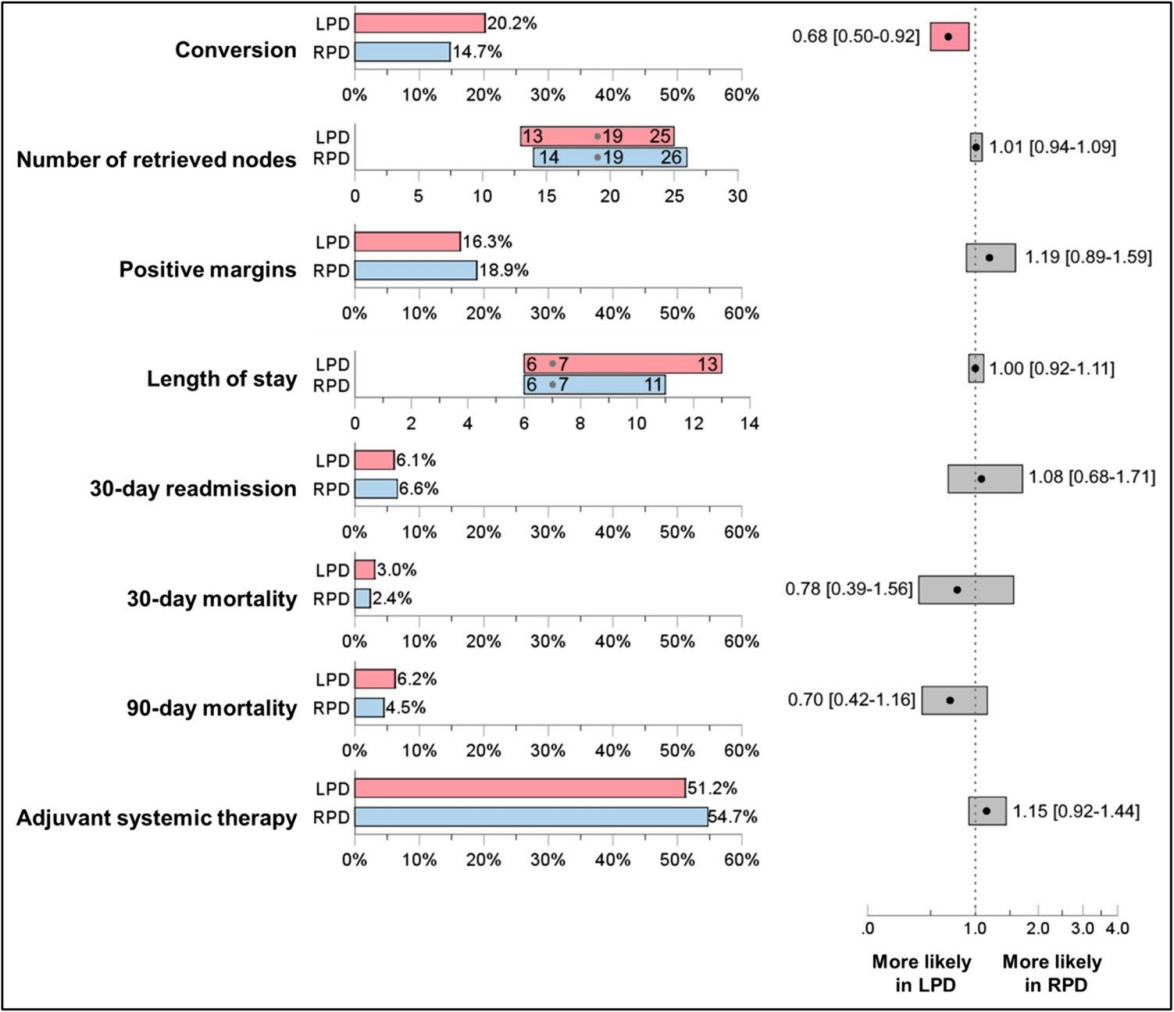
Comparison of short-term quality outcomes between the matched LPD and RPD groups

Multi-variate analysis of the PSM-cohorts demonstrated a similar rate of surgical and pathologic outcomes between groups (Fig. 2). Specifically, there was no difference between groups based on LOS (HR 1.00, 95%CI 0.92–1.11), 30-day readmission (HR 1.08, 95% CI 0.68–1.71), 30-day (HR 0.78, 95% CI 0.39–1.56) or 90-day mortality (HR 0.70, 95%CI 0.42–1.16), or ability to receive adjuvant therapy (HR 1.15, 95%CI 0.92–1.44). There was also no difference in the nodal harvest (HR 1.01, 95%CI 0.94–1.09) or the rate of positive histopathologic margin (HR 1.19, 95%CI 0.89–1.59). In fact, the only difference between groups was a higher rate of conversion to open in the LPD cohort (HR 0.68, *p* = 0.50–0.92).

**Fig. 3:**
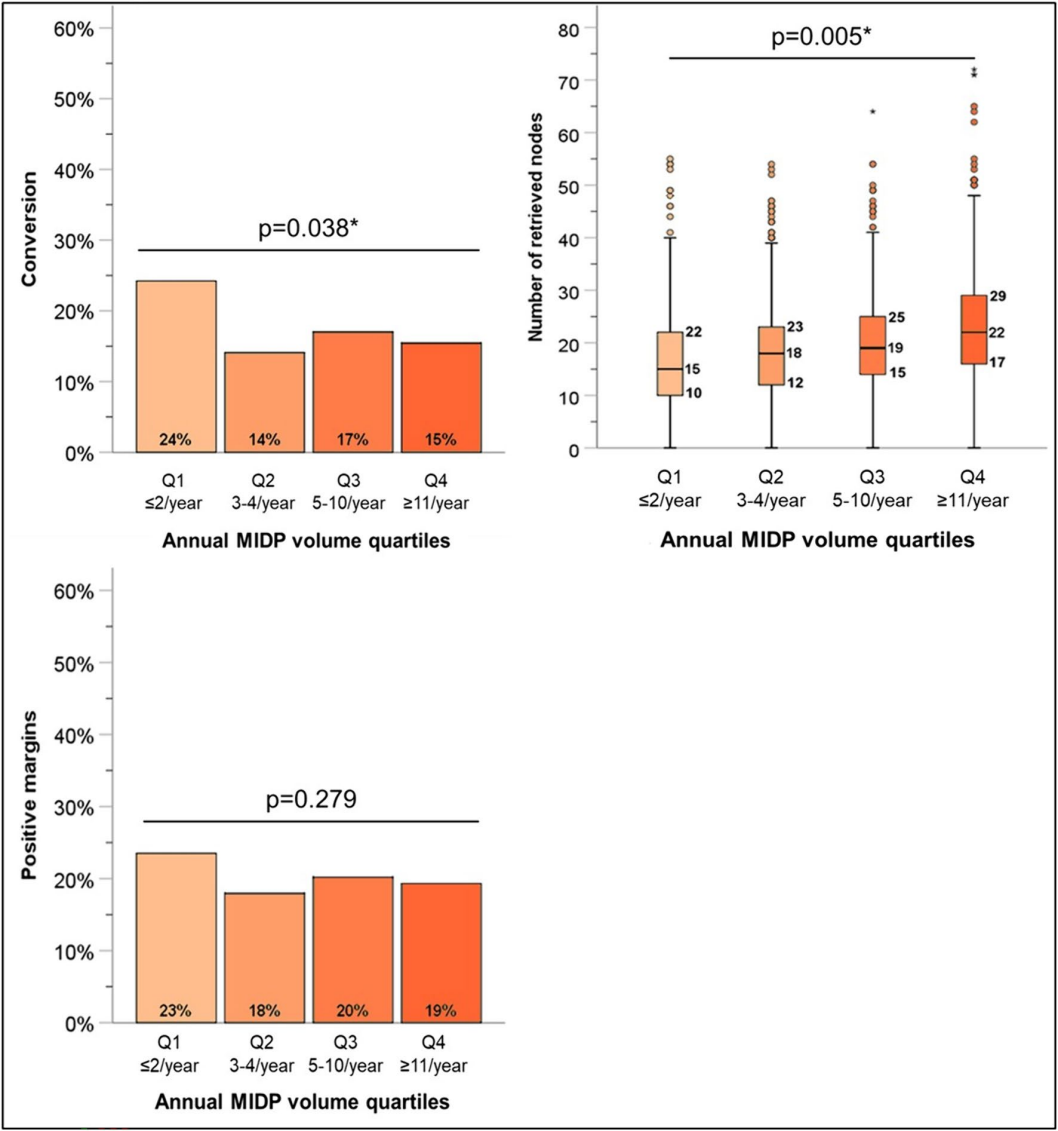
Impact of annual institutional MIPD volume on technical metrics. *MIPD* minimally invasive pancreatoduodenectomy. *Statistically significant

**Fig. 4:**
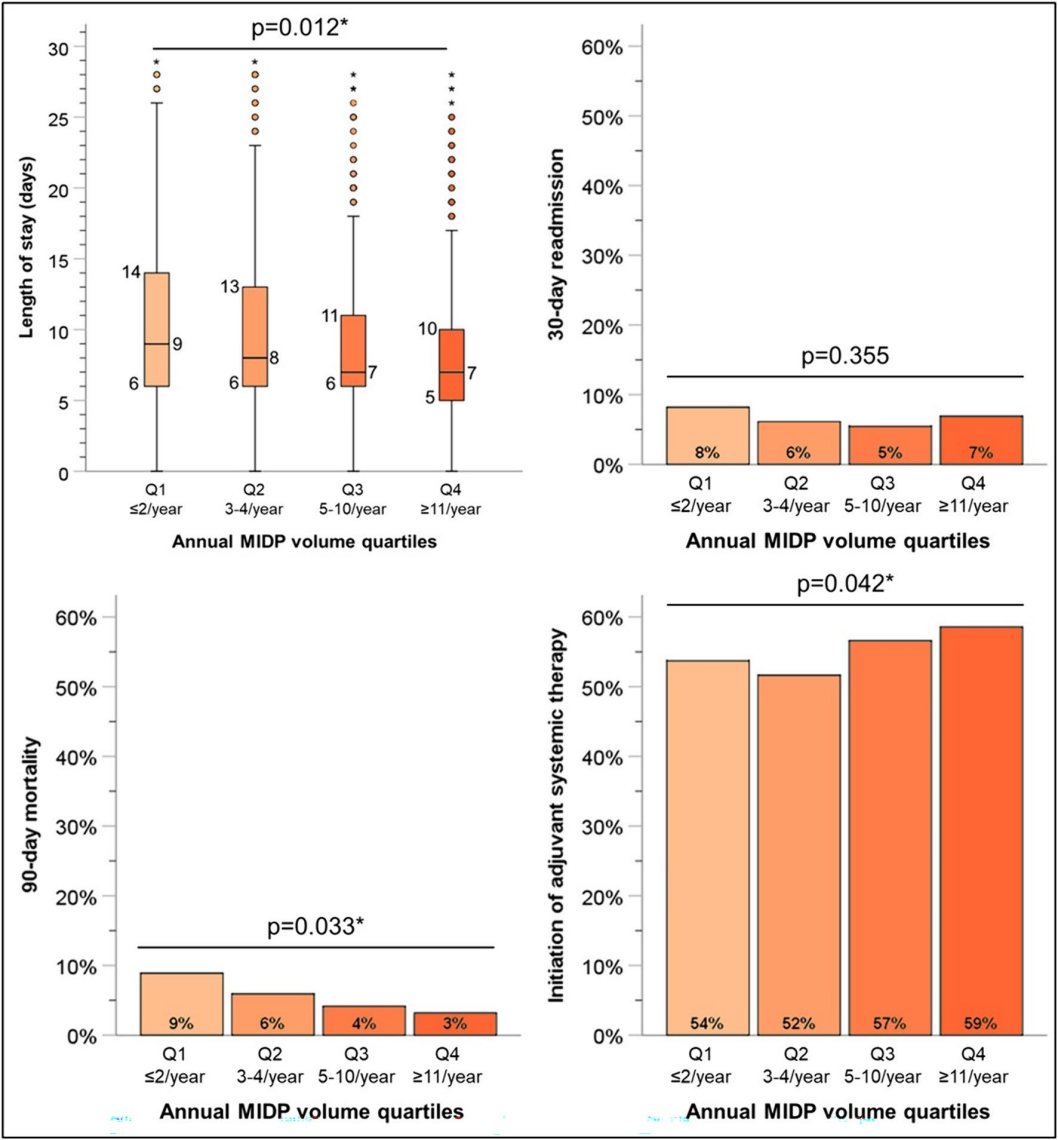
Impact of annual institutional MIPD volume on postoperative metrics. *MIPD* minimally invasive pancreatoduodenectomy. *Statistically significant

We next examined the impact of center-level annual case volume on outcomes, as the learning curve for minimally invasive pancreas surgery is known to be quite steep. Centers were divided into quartiles based on their annual case volume of MIPD (combined LPD and RPD) (Table 3). First, technical outcomes were assessed, demonstrating that lower annual vase volumes were associated with a lower number of retrieved lymph nodes (*p* = 0.005) and a higher rate of conversion to open (*p* = 0.038) (Fig. 3). Volume was also assessed for postoperative outcomes, showing that higher-volume centers had a shorter LOS (*p* = 0.012), higher rate of initiation of adjuvant therapy (*p* = 0.042), and, most strikingly, a reduction in 90-day mortality (*p* = 0.033) (Fig. 4).

**Table 3:** Annual center volume of MIPD by quartiles

	N of cases	N of centers	Mean annual MIPD volume	SD	95% CI	% of total centers in NCDB
Q1	881	313	0.93	0.51	[0.90–0.97]	70.7
Q2	932	82	3.16	0.74	[3.11–3.21]	18.5
Q3	774	36	7.24	1.85	[7.11–7.37]	8.1
Q4	755	12	24.44	12.63	[23.54–25.34]	2.7

## Discussion

This study is, to our knowledge, the largest to compare the laparoscopic and robotic Pancreatoduodenectomy, and the first to do so using propensity-matching to reduce confounding variable bias. We find that, when matched for potential confounders, the two approaches have similar rates of readmission, mortality, length-of-stay and completion of neoadjuvant therapy. We also find that the two approaches can achieve similar short-term oncologic success as measured by the nodal harvest and the rate of positive margins. We do conclude, like previous studies, that outcomes are best when performed in higher-volume centers, with improvement at a center-volume of at least 5 cases per year. [12, 13].

There has been a significant amount of data comparing open and minimally invasive pancreatectomy, with the general consensus that MIPD is, at least, a safe approach. Croome et al. showed that LPD was associated with a reduced LOS and an improved rate of initiation of adjuvant therapy versus OPD [14]. Stauffer et al. subsequently showed a similar survival rate, but with a greater nodal harvest in LPD vs OPD [15]. Subsequent analysis in large cohorts shows generally improved short-term outcomes with LPD, including reduced LOS at the cost of longer operative time; there is no reported difference in long-term oncologic outcomes between open and laparoscopic PD [16–19]. However, as case volumes in robotic surgery continue to rise, differences between laparoscopic and robotic approaches may influence the perceived outcomes of MIPD [20–22]. Previous smaller studies, such as Nassour et al. (2017), have found no difference in postoperative complication rates, but a reduced conversion rate with RPD [23–25]. The presented study represents this most modern cohort for this analysis and is the first propensity-matched analysis on the topic. Incorporating both perioperative and short-term oncologic outcomes, including initiation of adjuvant chemotherapy, which is often cited as a success metric in oncologic surgery, this study suggests that both RPD and LPD are technically appropriate and feasible approaches to this complex procedure based on provider comfort. This is particularly helpful in the setting of the recent LEOPARD trial demonstrating improved outcomes of the MIPD versus the open approach [26].

The concept of a “learning curve” in surgery, particularly with respect to minimally invasive surgery, has now been well established in impacting surgical outcomes [12, 27–29]. This is particularly true in pancreatic surgery, where outcomes are highly correlated with both personal and center-level case volumes [30–32]. However, given the more recent rise of MIPD, this concept is somewhat less studied in this cohort. Conroy et al. [33] and Adam et al. [34] both used larger databases to establish cut-offs of annual case-volumes associated with reduced complications, identifying 20 and 22 cases/year, respectively, as the target for improving outcomes [33, 34]. The former of these studies did utilize an older version of the NCDB for their analysis, while the Adam et al. article employed the National Inpatient Sample (NIS) up to 2012 [33]. Annual case volumes of RPD have nearly doubled from 2017 to 2020 compared with those prior to 2017, with RPD representing over 50% of MIPD cases in recent years (Fig. 1). Thus, by including more recent years, and confirming adequate representation of both RPD and LPD, we provide a modern, holistic assessment of the impact of learning curve on the outcomes of MIPD, confirming the findings of prior studies that higher case volumes do improve outcomes. While it is interesting that not all outcome metrics are volume-dependent, there is a clear trend toward improved technical and post-operative outcomes in high-volume centers. This could be considered a modifiable risk factor, wherein dedicated surgeons or surgical groups can place effort to improve patient care. We also found that improvement in outcomes happened upwards of 5 cases/year for the MIPD, which represented the upper two quartiles in analysis, and may represent an annual volume needed to progress along the learning curve. This is somewhat lower than the previously cited studies and shows that the target number is not so high as to unachievable by many centers nationwide [33, 34]. It is also notable that increasing use of the robotic platform does not eliminate the importance of experience. Thus, while robotic surgery is useful and exciting, it cannot replace diligent training or thoughtful repetition. We do want to note that this is a conceptual finding regarding surgeon experience, and there should not be a specific cut-off below which centers are considered “low volume”.

This study has limitations. Most notably are the inherent detriments of using large databases. While this allows us to increase sample size and generalizability, it also precludes detailed analyses of why a certain approach was chosen and may introduce confounding biases. We attempted to control for this by propensity-matching, but this cannot completely remove the potential for between-group bias. The NCDB does not record overall complication rates. Thus, mortality, readmission and initiation of adjuvant chemotherapy were employed as surrogate markers, but we cannot truly assess overall complication rates. Further, the NCDB, unlike NSQIP, does not include a granular report on postoperative morbidities like DGE, POPF, and many others that are pertinent to pancreatoduodenectomy. However, NCDB allows the aggregation of surgical cases by institutional codes to gauge annual volumes. NSQIP de-identifies this data which prohibits performing outcomes research based on institutional volumes. Moreover, NSQIP does not provide data on adjuvant therapies, and only recently started providing oncologic quality metrics like nodal harvest. After careful evaluation of both databases, we chose to go with NCDB which better serves the purpose of our clinical question, yet this then mandates that we employ length-of-stay, readmission rates, and mortality as general surrogates for the postoperative course. However, as mentioned, granular data regarding complication rates is not available, which is a limitation of this study. Finally, this study was not able to assess long-term oncologic outcomes, which may vary between groups. Short term-surrogates, including margin-positive resection, nodal harvest and initiation of adjuvant therapy were used in an attempt to address oncologic outcomes but this cannot be confirmed to translate into long-term equivalency.

## Conclusion

Laparoscopic and robotic Pancreatoduodenectomy have similar peri- and post-operative surgical and oncologic outcomes, with a lower rate of conversion to open in the robotic cohort. The robotic technique does.
